# Phospholipase D Is Involved in the Formation of Golgi Associated Clathrin Coated Vesicles in Human Parotid Duct Cells

**DOI:** 10.1371/journal.pone.0091868

**Published:** 2014-03-11

**Authors:** Lorena Brito de Souza, Luis Lamberti Pinto da Silva, Maria Célia Jamur, Constance Oliver

**Affiliations:** Department of Cell and Molecular Biology, Faculdade de Medicina de Ribeirão Preto, University of São Paulo, Ribeirão Preto, SP, Brazil; Institut Curie, France

## Abstract

Phospholipase D (PLD) has been implicated in many cellular functions, such as vesicle trafficking, exocytosis, differentiation, and proliferation. The aim of this study was to characterize the role of PLD in HSY cells, a human cell line originating from the intercalated duct of the parotid gland. As the function and intracellular localization of PLD varies according to cell type, initially, the intracellular localization of PLD1 and PLD2 was determined. By immunofluorescence, PLD1 and PLD2 both showed a punctate cytoplasmic distribution with extensive co-localization with TGN-46. PLD1 was also found in the nucleus, while PLD2 was associated with the plasma membrane. Treatment of cells with the primary alcohol 1-butanol inhibits the hydrolysis of phosphatidylcoline by PLD thereby suppressing phosphatidic acid (PA) production. In untreated HSY cells, there was only a slight co-localization of PLD with the clathrin coated vesicles. When HSY cells were incubated with 1-butanol the total number of clathrin coated vesicles increased, especially in the juxtanuclear region and the co-localization of PLD with the clathrin coated vesicles was augmented. Transmission electron microscopy confirmed that the number of Golgi-associated coated vesicles was greater. Treatment with 1-butanol also affected the Golgi apparatus, increasing the volume of the Golgi saccules. The decrease in PA levels after treatment with 1-butanol likewise resulted in an accumulation of enlarged lysosomes in the perinuclear region. Therefore, in HSY cells PLD appears to be involved in the formation of Golgi associated clathrin coated vesicles as well as in the structural maintenance of the Golgi apparatus.

## Introduction

The metabolism of phospholipids plays a key role in regulating intracellular vesicular transport and signal transduction. Phospholipase D (PLD) is a phospholipid-modifying enzyme that has been implicated in many cellular functions, such as vesicle coat recruitment, cytoskeletal rearrangement, vesicle budding from the Golgi apparatus and exocytosis [1]–[6]. PLD hydrolyses the terminal phosphodiester bond of phosphatidylcholine, the predominant membrane phospholipid, to produce phosphatidic acid (PA) and choline. PA is highly regulated in cells and can be converted to other potentially bioactive lipids, such as diacylglycerol and lysophosphatidic acid [7].

Two major mammalian isoforms of PLD have been identified, PLD1 [8] and PLD2 [9]. Both enzymes are widely expressed in a variety of tissues and cells [10], [11]. PLD1 and PLD2 have approximately 50% homology in the conserved catalytic core, and are more variable at the N- and C-termini [12], [13]. The catalytic core contains two HKD motifs that are responsible for enzymatic activity, the phox consensus sequence (PX) mediates protein-protein interactions or binds to phosphatidylinositol phosphates and the plekstrin homology (PH) domain determines the localization of the protein [7]. The intracellular distribution of PLD1 and PLD2 is controversial and the isoforms have been found in diverse organelles, such as, the Golgi apparatus, endosomes, nucleus, lysosomes, plasma membrane and endoplasmic reticulum [14]–[18]. The exact localization of endogenous PLD1 and PLD2 is difficult to determine because they are poorly expressed and the overexpressed tagged forms can result in an erroneous intracellular distribution of these proteins.

PLD has been identified in the Golgi apparatus and a role for PLD in vesicular trafficking in this organelle has been proposed [4], [15], [16], [19], [20]. It is possible that the PA produced by PLD can act as a structural lipid, recruiting coats and other necessary components for vesicle formation and budding in addition to promoting membrane curvature [21], [22]. Although PLD has been implicated in the secretion of amylase from acinar cells of salivary glands [2], there has been no study concerning the localization and role of PLD in vesicle trafficking in salivary gland duct cells. Therefore, the present study was undertaken in order to identify the intracellular distribution of the endogenous isoforms of PLD1 and PLD2 and to determine the role of PLD in the formation of vesicles from Golgi apparatus in intercalated duct cells of the parotid gland. The results demonstrate that PLD1 and PLD2 are present in the TGN (Trans Golgi Network) and distributed through the cytoplasm in salivary gland cells. In addition, PLD1 was present in the nucleus and PLD2 associated with the plasma membrane. Moreover, PLD appears to regulate the formation of clathrin-coated vesicles associated with Golgi apparatus as well as the morphological maintenance of Golgi apparatus and lysosomes in duct cells from the parotid gland.

## Materials and Methods

### Cells

HSY cells [23], generously provided by Dr. Indu Ambudkar (National Institute of Dental and Craniofacial Research, NIH, Bethesda, MD), were grown at 37°C in Dulbecco’s modified Eagle’s medium (DMEM) supplemented with 10% heat inactivated fetal calf serum, 100 U/mL penicillin and 100 mg/mL streptomycin (all from Life Technologies, Gibco, Grand Island, NY) in an humidified incubator with 5% CO_2_ in air.

### Treatments

Cells were treated with 1-butanol (1-ButOH), *tert*-butanol (*tert*-ButOH) (Merck Millipore, Darmstadt, Germany), brefeldin A (Sigma-Aldrich, St. Louis, MO), PLD1-specific inhibitor, (1*R*, 2*R*)-*N*-([S]-1-(4-5[5-bromo-2-oxo-2,3-dihydro-1H-benzo(d)imidazol-1-yl]piperidin-1-yl)propan-2-yl)-2-phenylcyclopropanecarboxamide [24] and PLD2-specfic inhibitor, *N*-(2-[4-oxo01-phenyl-1,3,8-triazasoiri(4,5)decan-8-yl]ethyl)quinolone-3-carboxamide [25] (Avanti Polar Lipids, Alabaster, AL) at 37°C for the indicated concentrations and times.

### Antibodies

The following primary antibodies were used: rabbit anti-PC specific PLD1 Internal (3 µg/mL, Life Technologies, Camarillo, CA), rabbit anti-PC specific PLD2 Internal (5 µg/mL, Life Technologies), mouse mAb anti-GM-130 (5 µg/mL, Clone 35/GM130, BD Transduction Laboratories, San Jose, CA), goat anti-clathrin HC (5 µg/mL, Santa Cruz Biotechnology Inc., Santa Cruz, CA) and sheep anti-human TGN46 (2 µg/mL, AbD Serotec, Oxford, UK). The following secondary antibodies were used for immunofluorescence: goat anti-mouse IgG F(ab)′_2_- Alexa 594, donkey anti-goat IgG F(ab)′_2_- Alexa 488, goat anti-rabbit IgG F(ab)′_2_- Alexa 594 and donkey anti-sheep IgG F(ab)′_ 2_- Alexa 633 (Life Technologies, Molecular Probes, Camarillo, CA).

### Immunofluorescence

Cells were cultured overnight on 13 mm round coverslips. The cells were rinsed in PBS, fixed for 15 min with 2% formaldehyde (Electron Microscopy Sciences, Fort Washington, PA) in PBS, rinsed and blocked for 30 min at room temperature in PBS containing 1% BSA (Sigma-Aldrich). The cells were then permeabilized for 10 min with 0.3% Triton X-100 (Sigma-Aldrich) in PBS, rinsed and labeled with the primary antibody diluted in PBS containing 1% BSA for 1 h at room temperature. After incubation, the cells were rinsed thoroughly in PBS and the samples were incubated for 30 min at room temperature with the secondary antibody diluted in PBS. The cells were then rinsed in PBS, coverslips mounted with Fluoromount-G (Electron Microscopy Sciences) and examined with a Leica TCS-SP5 scanning confocal microscope (Leica Microsystems, Heidelberg, Germany).

### Transmission Electron Microscopy (Tem)

Cells were plated in 6 well tissue culture plates and cultured for 3 days. Media was changed 16 h prior to fixation. Cells were fixed in 2% glutaraldehyde plus 2% formaldehyde (Electron Microscopy Sciences) in 0.1 M cacodylate buffer, pH 7.4 containing 0.05% CaCl_2_ for 1 h at room temperature. Cells were post-fixed in 1% OsO_4_ (Electron Microscopy Sciences) in 0.1 cacodylate buffer, pH 7.4, for 2 h, rinsed in Milli-Q water (Millipore, Billerica, MA) and dehydrated in a graded series of ethanol. Cells were removed from the tissue culture plates with propylene oxide and embedded in EMBED 812 (Electron Microscopy Sciences). Thin sections were cut with a diamond knife, mounted on copper grids and stained for 10 min each in Reynold’s lead citrate [26] and 0.5% aqueous uranyl acetate and examined with JEOL JEM-100CX II transmission electron microscope (Jeol Ltda., Tokyo, Japan).

### Lysotracker Staining

The acid vesicles were labeled with LysoTracker DND-99 (Life Technologies, Molecular Probes) using cells cultured overnight on 35 mm glass bottom culture dishes (MatTek Corporation, Ashland, MA). The cells were rinsed in PBS, incubated with 75 nM of LysoTracker DND-99 in DMEM for 30 minutes. The cells were rinsed with PBS and treated or not with 0.5% 1-butanol or 0.5% *tert-*butanol. The cells were examined with a Leica TCS-SP5 scanning confocal microscope (Leica).

### Plasmids and Transient Transfections

For transfection, cells were plated and transfected the next day using with a plasmid encoding CI-M6PR-GFP and Lipofectamine 2000 (Life Technologies). Experiments were performed 24 h after transfection.

### Image Analysis

Quantification of the volume of the Golgi apparatus and acid vesicles was done as previously described (Radulescu et al., 2007). All the experiments were performed in triplicate and 20–40 cells were considered for each experiment. Z-series images were quantified using ImageJ (National Institutes of Health, http://rsb.info.nih.gov/ij/). Regions of interest (ROIs) through the Z-series corresponding to the Golgi apparatus and acid vesicles were generated by thresholding GM-130 and Lysotracker staining, respectively. The ROI volume was determined by the total number of pixels in the ROIs through series. The volume reconstruction of the acid vesicles was made using ImageJ.

For measurement and fluorescence intensity, the ROIs corresponding to the threshold of GM-130, clathrin, CI-M6PR-GFP and TGN-46 fluorescence were quantified using ImageJ. The ROI area and fluorescent intensity were determined by the total number of pixels in the ROIs through series. For colocalization quantification, ImageJ was used to calculate the area of the region of overlap between two fluorescent probes and their total area as well. The area overlap for the anti-TGN46 × anti-PLD1, anti-TGN46 × anti-PLD2– labeled structures were reported as percentage of the anti-TGN46 total area.

## Results

### PLD1 and PLD2 Show Differences in their Intracellular Localization

**Figure 1:**
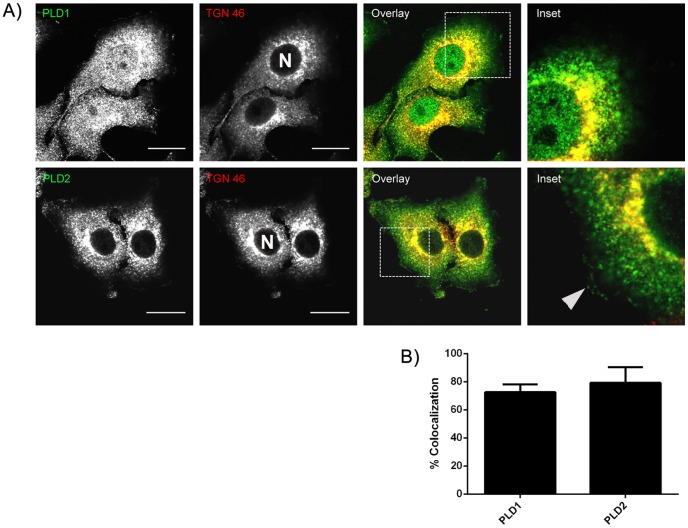
PLD1 and PLD2 are present in the Trans-Golgi Network. A) Cells were immunostained for PLD1 and PLD2 (green) and TGN-46 (red). Both isoforms of PLD have a punctuate distribution throughout the cytoplasm. PLD1 is highly expressed in the nucleus (N, nucleus) and PLD2 is associated with the plasma membrane (Inset, arrowhead). The perinuclear distribution of TGN-46 co-localized with PLD1 and PLD2. Images are single focal planes. Bar, 16 µm. B) Quantification of the colocalization between TGN and PLD1 and TGN and PLD2. Data is expressed as % colocalization±SEM from 3 experiments.

Since the intracellular localization and function of PLD varies according to the cell type [15], [17], [18], [27], initially, the localization of endogenous PLD isoforms in HSY cells was determined. By immunofluorescence, PLD1 and PLD2 exhibited a punctuate distribution in the cytoplasm. In addition, PLD1 showed an intense nuclear staining (Figure 1A) while PLD2 was localized to the plasma membrane (Figure 1A, inset, arrowhead). In order to determine if PLD1 and PLD2 were both associated with a particular organelle, immunofluorescence for PLD1 or PLD2 and TGN-46 (TGN marker) was performed. 72.55% ±3.239 of TGN-46 co-localized with PLD1 and 79.25±6.489 of TGN-46 co-localized with PLD2. (Figure 1B), indicating that both PLD isoforms are present in the TGN in HSY cells.

### PLD Activity is Involved in the Structural Maintainance of the Golgi Apparatus

**Figure 2:**
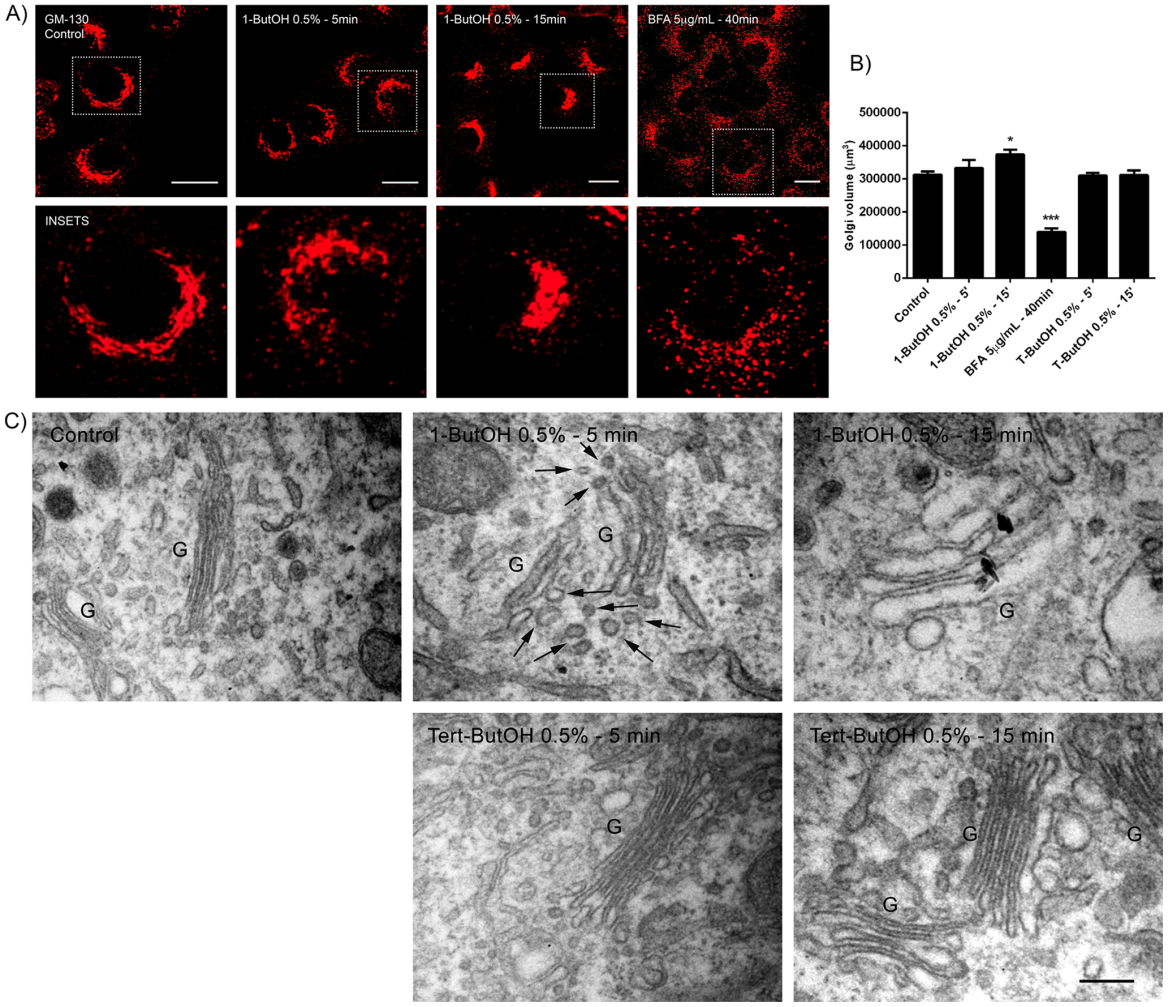
Treatment with 1-ButOH alters the morphology of the Golgi apparatus. A) Cells were untreated or treated with 0.5% 1-ButOH or 5 µg/mL BFA for the indicated times and the Golgi apparatus immunostained for GM-130. With treatment with 1-ButOH, the Golgi apparatus becomes disorganized and the saccules are dilated, while the treatment with BFA resulted in a vesiculation and spreading of the Golgi apparatus. Images are Z-series projections. Bar, 16 µm. B) Quantification of the Golgi volume by pixel intensity of GM-130 staining-thresholded areas. After 15 min of incubation with 1-ButOH there is a significant increase in the volume of the Golgi apparatus (p<0.05, Dunnett’s Multiple Comparison Test). C) Ultrastructural analysis shows control untreated cells with a characteristic Golgi apparatus composed of stacks of flattened cisternae. In cells treated with 0.5% 1-ButOH for 5 min the Golgi saccules are slightly swollen and by 15 min the Golgi apparatus is disorganized and the saccules are dilated. Numerous coated vesicles (arrows) are evident in close proximity to the swollen cisternae after 5 min of treatment with 0.5% 1-ButOH. Treatment with 0.5% *tert-*ButOH for 5 min or 15 min does not modify the structure of Golgi apparatus. (G, Golgi apparatus). Bar, 500 nm.

It has been established that in some cell types, PLD is associated with and has a functional involvement with the Golgi apparatus [21], [28]. Therefore, it was of interest to examine the role of PLD in the Golgi apparatus in HSY cells. Cells were treated with 1-ButOH or *tert*-ButOH for 5 and 15 minutes and then immunostained with GM-130 antibody (Figure 2). The untreated control cells exhibited the characteristic lace-like distribution of the Golgi apparatus while in 1-ButOH treated cells, the organization and architecture of the Golgi apparatus was altered. The Golgi apparatus was more compact than in the control cells and the saccules were dilated, so that they appeared as condensed vesicular structures. These characteristics were most evident in the cells treated with 1-ButOH for 15 minutes (Figure 2A). As expected, in cells treated with *tert*-ButOH, which does not interfere with the production of PA by PLD, the Golgi apparatus was similar to control cells (data not shown).

It is known that brefeldin A (BFA), a fungal metabolite that inhibits the guanine nucleotide exchange factor (GEF) of Arf-1, causes a reversible rearrangement of the membrane components of Golgi apparatus through the cytoplasm. In order to compare the modified profiles in the Golgi apparatus produced by 1-ButOH and BFA treatment, the cells were treated with 5 µg/ml BFA for 40 min and immunostained with GM-130. In cells treated with BFA, the Golgi apparatus was vesiculated and dispersed throughout the cytoplasm, in contrast to the compact and dilated saccules of the Golgi apparatus from cells treated with 1-ButOH (Figure 2A). These results show that the role of PLD in maintaining the structure of the Golgi apparatus is different than that of Arf-1. The relative increase in the volume of the Golgi apparatus when the cells were treated with 1-ButOH was confirmed by quantifying the volume of the organelle after immunostaining with anti-GM-130 in untreated and cells treated with BFA, 1-ButOH and *tert*-ButOH. Indeed, 1-ButOH treatments caused an increase in the volume of the Golgi apparatus that was more prominent in the cells treated with 1-ButOH for 15 minutes (Figure 2B).

To analyze in more detail the morphological changes that occur in the Golgi apparatus after 1-ButOH treatments, the cells were examined by transmission electron microscopy (TEM). Control untreated cells exhibited a typical well organized Golgi apparatus composed of flattened cisternae. In contrast, cells treated with 1-ButOH had a disorganized Golgi apparatus accompanied by a swelling of the Golgi saccules. After incubation with 1-ButOH for 15 minutes, virtually all of the Golgi cisternae were swollen (Figure 2C). These results confirmed the findings by immunofluorescence and quantification of the volume of the Golgi apparatus. Surprisingly, the number of coated vesicles associated with the Golgi apparatus apparently increased after 5 minutes incubation with 1-ButOH (Figure 2C, arrows). The cells treated with *tert*-ButOH did not exhibit any augmentation of the Golgi saccules or any increase in the number of coated vesicles, and had morphology similar to that observed in the control cells (Figure 2C).

**Figure 3:**
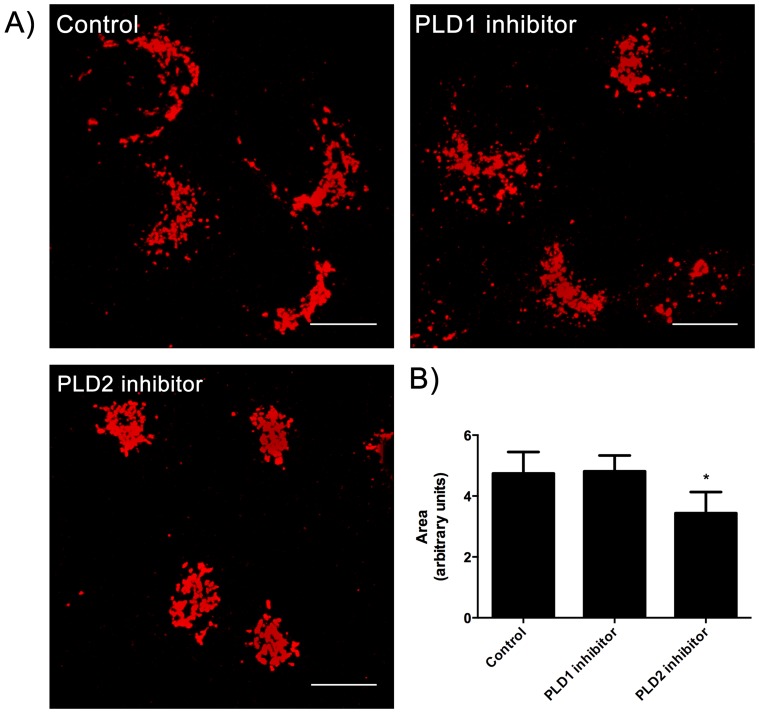
Treatment with specific PLD2 inhibitor alters the morphology of the Golgi apparatus. A) Cells were untreated or treated with 1 µM of PLD1 inhibitor or PLD2 inhibitor for 30 min and the Golgi apparatus immunostained for GM-130. The treatment with PLD2 inhibitor alters the organization of the Golgi apparatus and it becomes more compact. Treatment with PLD1 inhibitor did not affect the morphology of Golgi apparatus which is similar to the untreated cells. The images are Z-series projections. Bar, 10 µm. B) Quantification of the Golgi area by GM-130 staining-thresholded areas. The PLD2 inhibitor treatment significantly decreases the area of the Golgi apparatus when compared to untreated cells (p<0.05, Dunnett’s Multiple Comparison Test).

Since 1-ButOH is not specific for a given PLD isoform and inhibits the production of PA by both PLD1 and PLD2, it was essential to know which PLD isoform is involved in the maintenance of the morphology of the Golgi apparatus in HSY cells. Cells were treated with 1 µM of PLD1-specific inhibitor or PLD2-specific inhibitor for 30 min and then immunostained with anti-GM-130 to analyze the morphology of the Golgi apparatus. The morphology of the Golgi apparatus of the cells treated with PLD1 inhibitor is very similar to the untreated cells (Figure 3A). However, in cells treated with the PLD2 specific inhibitor the Golgi was more compact (Figure 3A). Quantification of the area occupied by the Golgi apparatus confirmed these differences (Figure 3B).

Taken together, these data demonstrate that PLD, particularly PLD2, has a role in the structural maintenance of Golgi apparatus. The decrease of PA level due the 1-ButOH treatment and incubation with PLD2-specific inhibitor results in a change in the morphology of the Golgi apparatus and an apparent increase in the number of Golgi associated coated vesicles in HSY cells.

### PLD Appears to be Involved in the Formation of Golgi Associated Clathrin-coated Vesicles

**Figure 4:**
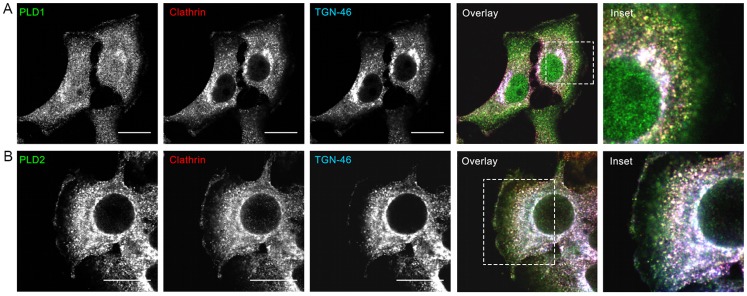
Co-localization between PLD1, PLD2, clathrin and TGN-46 in HSY cells. PLD1 and PLD2 (green) are distributed in the cytoplasm and concentrated in the juxtanuclear region, where they are extensively co-localized with clathrin (red) and TGN-46 (blue). Images are single focal planes. Bar, 16 µm.

Previous studies have shown that treatment of rat pituitary cells (GH3) cells with 1-ButOH inhibits the budding of nascent vesicles from the Golgi apparatus [1], [19]. The increased number of Golgi associated coated vesicles, when HSY cells were treated with 1-ButOH (Figure 2C), suggests that PLD may have a role in the formation of clathrin-coated vesicles (CCVs) derived from the Golgi. To investigate the association of PLD with the formation of CCVs, the localization of PLD1 and PLD2 with CCVs and TGN was analyzed. To visualize the CCVs, the cells were immunostained using an antibody against the heavy chain of clathrin. Following immunostaining, clathrin can be seen associated with the plasma membrane and extensively co-localized with PLD1 and PLD2 in the TGN region. There was little co-localization of clathrin and the PLD isoforms adjacent to the plasma membrane (Figure 4).

**Figure 5:**
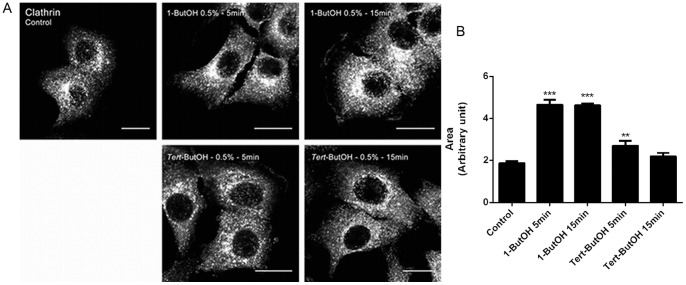
Inhibition of PA production alters the distribution of clathrin. Cells were untreated or treated with 1-ButOH and *tert-*ButOH for the indicated times and labeled using an anti–clathrin antibody. In control untreated cells, clathrin is dispersed throughout the cytoplasm in a punctuate manner. After the treatment with 1-ButOH for 5 min and 15 min, the intracellular distribution of clathrin was changed, concentrating in the perinuclear region, however the treatment with *tert*-ButOH did not alter the intracellular distribution of clathrin. All micrographs are projected Z-series images. Bar, 16 µm. B) Quantification of clathrin pixel intensity in perinuclear-thresholded areas (p<0.05, Dunnett’s Multiple Comparison Test).

To address the question whether PLD plays a role in the formation of Golgi associated CCVs, HSY cells were incubated with 1-ButOH for 5 and 15 min. Following treatment with 1-ButOH, clathrin had changed its intracellular distribution, concentrating in the perinuclear region. In contrast, the treatment with *tert-*ButOH had no effect on the distribution of clathrin (Figure 5A). Quantification of the area of clathrin staining in perinuclear area demonstrated that clathrin accumulated in the perinuclear region after 1-ButOH treatments (Figure 5B), indicating that PLD may regulate the formation of CCVs from Golgi apparatus in HSY cells.

### The Role of PLD in the Trafficking of Proteins Transported by CCV

CCVs are involved in the trafficking of many proteins to the plasma membrane, endosomes and lysosomes [29], [30]. To investigate the role of PLD in the trafficking of proteins dependent on CCVs, the distribution of TGN-46 and cation-independent mannose-6-phosphate receptor (CI-M6PR) were analyzed after 1-ButOH and *tert-*ButOH treatments for 5 and 15 min. TGN-46 transits between the TGN and the plasma membrane and CI-M6PR is responsible for transporting acid hydrolases from the Golgi apparatus to lysosomes. Both proteins traffic via CCVs.

**Figure 6:**
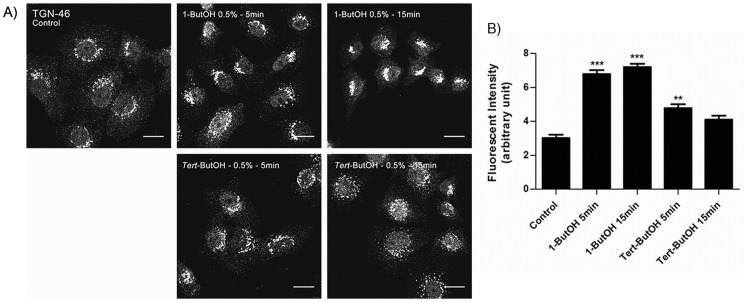
PLD has a role in TGN-46 trafficking. Cells were untreated or treated with 1-ButOH and *tert-*ButOH for the indicated times and immunostained using TGN-46 antibody. In control untreated cells, TGN-46 is associated with the plasma membrane and concentrated in the perinuclear region. After the treatment with 1-ButOH, TGN-46 is no longer associated with the plasma membrane and is concentrated in the perinuclear region. Cells treated with *tert-*ButOH have the same characteristics of control cells. All micrographs are projected Z-series images. Bar, 16 µm. B) Quantification of TGN-46 pixel intensity in perinuclear-thresholded areas (p<0.05, Dunnett’s Multiple Comparison Test).

In control untreated cells, TGN-46 was associated with the plasma membrane and was localized in perinuclear region. After treatment with 1-ButOH, TGN-46 concentrated in the perinuclear region and absent from the plasma membrane. The treatment with *tert*-ButOH did not change the distribution of TGN-46 (Figure 6A). Fluorescence intensity quantification of TGN-46 confirmed the immunofluorescence results that TGN-46 was concentrated in the perinuclear region after 1-ButOH treatments (Figure 6B), suggesting that PLD activity may regulate the trafficking of TGN-46 to the plasma membrane.

**Figure 7:**
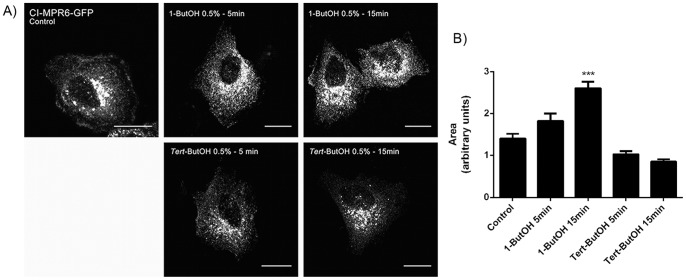
PLD regulates CI-M6PR trafficking. HSY cells were transfected with CI-M6PR-GFP. Transfected cells were untreated or treated with 1-ButOH and *tert-*ButOH for the indicated times. In control untreated cells, CI-M6PR-GFP was distributed throughout the cytoplasm, but concentrated in the perinuclear region. After the treatment with 1-ButOH, CI-M6PR-GFP concentrated extensively in the perinuclear area. Cells treated with *tert-*ButOH have the same characteristics of control cells. All micrographs are projected Z-series images. Bar, 16 µm. B) Quantification of CI-M6PR area in perinuclear-thresholded areas (p<0.05, Dunnett’s Multiple Comparison Test).

Examination of CI-M6PR-GFP localization demonstrated that in control untreated cells, CI-M6PR-GFP was present in the perinuclear region and after the treatment with 1-ButOH, the protein accumulated in this region in a time-dependent manner. Cells treated with *tert*-ButOH, CI-M6PR-GFP showed the same distribution as control cells (Figure 7A). Quantification of the area occupied by the CI-M6PR-GFP indicated that the protein accumulated in the perinuclear area after 1-ButOH treatments (Figure 7B), suggesting that PLD activity also regulates the trafficking of CI-M6PR from the Golgi apparatus to endosomes.

**Figure 8:**
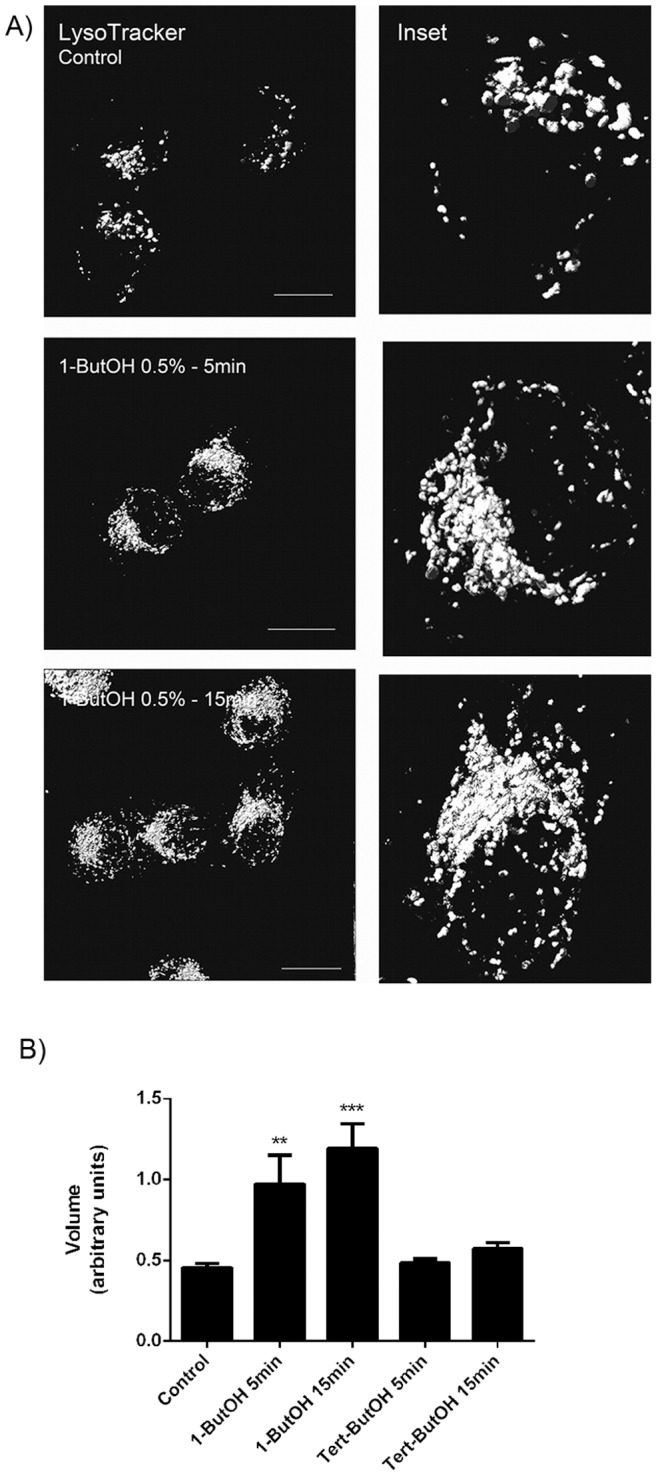
The decrease in PA levels resulted in an accumulation of lysosomes in the juxtanuclear region. A) Cells were labeled with LysoTracker and then untreated or treated with 1-ButOH for the indicated times. The treatment with 1-ButOH resulted in an increase in the number of lysosomes in the juxtanuclear region. After 15 min of treatment, the lysosomes seem to be fused with each other. The figures are volume reconstructions of the micrographs following LysoTracker staining. Bar, 16 µm. B) Quantification of CI-M6PR volume by pixel intensity of Lysotracker staining-thresholded areas (p<0.05, Dunnett’s Multiple Comparison Test).

The disturbance in the CI-M6PR trafficking after 1-ButOH treatment could also affect the traffic of acid hydrolases to the lysosomes and consequently the degradation of macromolecules inside this organelle. To investigate the relationship between PLD activity and lysosomes, HSY cells were stained with Lysotracker and treated with 1-ButOH and *tert-*ButOH. In control untreated cells, the lysosomes were distributed close to the perinuclear region. After 1-ButOH treatment, the lysosomes were larger and the area occupied by the lysosomes appeared to increase (Figure 8A). Volume quantification of the lysosomes confirmed that there was an increase in the average volume of the lysosomes after 1-ButOH treatment (Figure 8B). These results suggest that the PA level decrease due 1-ButOH treatment can produce a volume augmentation in the lysosomes most likely due to impairment in the degradation of macromolecules which accumulate inside the organelle.

## Discussion

This study demonstrated that PLD1 and PLD2 were present in the TGN and distributed through the cytoplasm in salivary gland cells. In addition, PLD1 was present in the nucleus and PLD2 was associated with the plasma membrane. It was also shown that PLD activity was important for the formation of clathrin-coated vesicles associated with Golgi apparatus and in the morphological maintenance of the Golgi apparatus and lysosomes.

In agreement with previously published results, PLD1 and PLD2 exhibited a punctuate cytoplasmic distribution and were present in the perinuclear region in HSY cells. Furthermore, PLD1 showed a nuclear staining and PLD2 was present in the plasma membrane. The same localization of PLD1 has also been reported for GH3 cells, HEK cells and pig vascular smooth muscle cells [15], [18], [31], [32]. Gayral *et al* (2006) have shown that the activity of PLD1 in the nucleus is related with the metabolism of nuclear phospholipids for the activation of PKC and ERK that are responsible for cellular proliferation. The plasma membrane localization of PLD2 has also been seen in NRK cells, NIH 3T3 cells, mouse adipocytes and cardiomyocytes [16], [17], [33]. It was demonstrated that PLD2 in the plasma membrane modulates endocytosis of certain signaling receptors [17], [33], [34], as well as the recycling of transferrin receptor [35].

The perinuclear localization of PLD1 and PLD2 is most likely related to their function in the salivary gland cells. The presence of the isoforms in the TGN and the alteration in the morphology of Golgi apparatus with 1-ButOH treatment, which inhibits PA production by PLD, indicate that these enzymes may play an important role in salivary gland cells. PLD is capable of performing a transphosphatidylation reaction using a primary alcohol, such as 1-ButOH to generate phosphatidylbutanol, instead of PA. This transphosphatidylation reaction is a highly specific to PLD and has been used widely to assay PLD activity as well as to demonstrate the requirement of PA produced by PLD. In PLD2 knockdown experiments, the Golgi apparatus becomes totally disorganized and vesicles associated with the Golgi apparatus accumulate in the lateral portions of the organelle [4]. GH3 cells treated with 1-ButOH also show an alteration in the morphology of Golgi apparatus [19]. It is possible that the decrease in PA can impair the fission of Golgi associated coated vesicles. These vesicles would remain attached to the Golgi saccules contributing to the enlargement of the Golgi apparatus. Perhaps, the increased number of coated vesicles in the Golgi apparatus seen by TEM when the cells were treated with 1-ButOH for 5 min may be vesicles whose fission from the Golgi saccules has been arrested and because of their orientation in the section they appear to be free in the cytoplasm. RBL-2H3 cells that overexpress the catalytic inactive form of PLD2 (PLD2CI cells) have a disorganized Golgi apparatus with dilated cisternae and an increase in associated vesicles. The addition of PA in these cells resulted in the restoration of Golgi phenotype in PLD2CI cells [36]. Previous biochemical and morphological studies have identified an association of PLD with the Golgi apparatus [14]–[16] as well as its role in the function of this organelle, especially in the TGN [1], [4], [20]. Chen et al. (1999) also demonstrated that PA produced by PLD is necessary for vesicle release from the TGN in GH3 cells. The modification in the morphology of Golgi apparatus due the 1-ButOH treatment was opposite from BFA treatment. Therefore, it suggests that PLD has a role in the maintenance of Golgi apparatus different from others proteins, like ARF-1.

Although previous studies have shown that PLD has a role in the formation of vesicles produced in the TGN, there is no study showing that these vesicles could be CCVs. The coated vesicles that are released from the TGN are coated with either COPI or clathrin [37]. The present study demonstrated that clathrin was concentrated in the perinuclear region in HSY cells treated with 1-ButOH. Additionally, previous studies have shown that depletion of PLD by small interfering RNA (siRNA) increases COPI vesicles at the rims of Golgi and results in a swelling of the organelle [4].

Since the PLD activity is important for the formation of CCV associated with the Golgi apparatus, this enzyme could also regulate the intracellular traffic of other proteins that utilize CCV. PLD1 and PLD2 have been shown to be involved in the trafficking of PTH type 1 (Parathyroid Hormone Type 1) receptor in CHO and ROS cells [38]. The inhibition of PLD activity with 1-ButOH treatment or PLD knockdown also impairs the traffic of the BCR (B cell receptors) in B lymphocytes. In parotid gland cells, PLD regulated the trafficking of the CCV-dependent proteins, TGN-46 and CI-M6PR. The present study shows that when HSY cells were treated with 1-ButOH, TGN-46 became concentrated in the perinuclear region and was no longer associated with the plasma membrane. This suggests that in the absence PA produced by PLD TGN-46 was retained in the TGN impairing its trafficking to plasma membrane. CI-M6PR also had its trafficking affected when PA production by PLD was impaired. CI-M6PR is a receptor that carries acid hydrolases from the TGN to lysosomes. In the lysosomes, acid hydrolases dissociate from CI-M6PR receptor and act to degrade macromolecules. Subsequently, CI-M6PR returns to TGN to be reused in the trafficking of other acid hydrolases to the lysosomes [39]–[41]. The 1-ButOH treatment resulted in the concentration of CI-M6PR in the perinuclear region and the loss of this protein from cytoplasmic vesicles. Therefore, PLD may also regulate the CI-M6PR trafficking and consequently the trafficking of acid hydrolases to lysosomes in HSY cells.

The morphology and distribution of lysosomes was also dependent on PLD activity in salivary gland cells. After 1-ButOH treatment, lysosomes concentrated in the perinuclear region and became enlarged in HSY cells. It is possible that the decrease in PA production by PLD and, consequently, the impaired trafficking of CI-M6PR and acid hydrolases to the lysosomes could cause an increase in the volume of these organelles. In Hela cells, knockdown of GARP (Golgi-associated Retrograde Protein complex) impaired the trafficking of CI-M6PR to the TGN and increased the volume of lysosomes. CI-M6PR accumulated in a vesicle population and did not go to the TGN. Therefore, the acid hydrolases did not translocate to lysosomes, and they became larger due to the accumulation of non-degraded macromolecules [30].

The mechanism by which PLD and PA regulate vesicle formation from cellular membranes is unknown. A previous study suggests that PLD acts like a GAP protein, through its phox domain, activating dynamin and allowing the fission of clathrin-coated vesicles from the plasma membrane [42]. PA can also recruit coat and other proteins required for vesicle formation, such as COPI, kinesin and ARF [43], [44]. The conical shape of PA results in a negative curvature of membranes that helps in the fission of cellular membranes [21], [45]–[47]. PLD and PA have binding partners, such as phosphoinositides, small GTPases, phosphatases and kinases [22] and the role of PLD and PA in vesicle formation is due their physicochemical properties and, protein-protein and lipid-protein interactions.

In summary, the findings of the present study indicate that the PLD activity more specifically, PLD2, has an apparent role in the formation of clathrin-coated vesicles from Golgi apparatus and TGN, as well as, in the structural maintenance of the Golgi apparatus and lysosomes in HSY cells. Additional studies are necessary in order to establish how PLD regulates the formation of CCVs from Golgi apparatus. These additional studies may elucidate new pathways that regulate secretion in salivary gland cells.
